# Exosomal circ-0020887 and circ-0009590 as novel biomarkers for the diagnosis and prediction of short-term adverse cardiovascular outcomes in STEMI patients

**DOI:** 10.1515/med-2023-0807

**Published:** 2023-10-11

**Authors:** Guan Wang, Chun Wang, Zhengyi Huang, Shuai Sun, Yanjun Chen

**Affiliations:** Department of Cardiology, Peking University Shenzhen Hospital, Shenzhen, 518036, China; Department of Geriatrics, Shenzhen Longhua District Central Hospital, Shenzhen, 518110, China; Department of Cardiology, Peking University Shenzhen Hospital, No. 1120, Lianhua Road, Futian District, Shenzhen, 518036, China

**Keywords:** STEMI, exo-circ-0020887, exo-circ-0009590, MACE-free 1-year survival rate, biomarkers

## Abstract

This study attempted to identify exosomal circular RNAs (circRNAs) as diagnostic and prognostic biomarkers for patients with ST-segment elevation myocardial infarction (STEMI). The differentially expressed exosomal circRNAs (DEECs) were screened from microarray dataset (GSE160717 and GSE197137) and RNA-Seq dataset (GSE208194), and the expression levels of DEECs in patients with STEMI were validated using reverse transcription and quantitative real-time PCR. The diagnostic value of DEECs was assessed using receiver operating characteristic curves. The major adverse cardiovascular event (MACE)-free 1-year survival rate was evaluated using the Kaplan–Meier method, and the factors affecting prognosis were determined using Cox regression model analysis. Results showed that four DEECs were screened including exo-circ-0001490, exo-circ-0020887, exo-circ-0009590, and exo-circ-0055440, and only upregulated exo-circ-0020887 and exo-circ-0009590 expression was validated in patients with STEMI. The exo-circ-0020887 and exo-circ-0009590 expression was positively correlated with hs-CRP, LDL-C, cTnI, and CK-MB. The exo-circ-0020887 and exo-circ-0009590 showed good diagnostic efficacy to distinguish STEMI patients from healthy controls (area under the curves: 0.85 and 0.80). STEMI patients with high levels of exo-circ-0020887 and exo-circ-0009590 had lower MACE-free 1-year survival rate, and exo-circ-0020887 and exo-circ-0009590 expression was independent risk factors for adverse prognosis. In summary, upregulation of plasma exo-circ-0020887 and exo-circ-0009590 might act as potential biomarkers for the diagnosis and prediction of short-term adverse cardiovascular outcomes in patients with STEMI.

## 1 Introduction

ST-segment elevation myocardial infarction (STEMI) is a serious form of acute coronary syndrome, which mainly caused by acute thrombotic coronary artery occlusion at the site of a ruptured pre-existing atherosclerotic plaque [1]. At present, STEMI remains a significant contributor to the high rate of morbidity and mortality worldwide [2]. Over the past few decades, several conventional biomarkers including lactate dehydrogenase, cardiac troponin I (cTnI), creatine kinase isoenzymes (CK-MB), and myoglobin have been used as diagnostic biomarkers for STEMI [3]. However, the application of these biomarkers in the early stage of STEMI onset is limited due to low sensitivity, and there is a lack of prognostic biomarkers for STEMI. Therefore, identification of novel biomarkers with high specificity and sensitivity for STEMI is urgently needed.

Exosomes are first reported by Trams EG team in 1981, which are small extracellular vesicles at 40–150 nm in diameter and contain a specific cargo of proteins, mRNAs, and non-coding RNAs (ncRNAs) [4]. Exosomes are secreted by a wide variety of eukaryotic cells and are existed in nearly all body fluids, including plasma, serum, sweat, tears, saliva, urine, breast milk, gastric juice, and cerebrospinal fluid [5,6]. Recently, exosomes are found to functionally modulate the activities of neighboring and distant recipient cells and participate in the regulation of various pathological states in human diseases [7,8]. Circular RNAs (circRNAs) are a special type of endogenous ncRNAs, which characterized by covalently closed loop structures and highly resistant to Ribonuclease R (RNase R) activity [9]. It has been suggested that circRNAs are enriched and stable in exosomes. Currently, with the development of gene microarray and sequencing, a large number of dysregulated exosomal circRNAs have been found in serum and plasma [10]. Accumulating evidence has indicated that circRNA can be encapsulated in exosomes and participate in the pathological process of various diseases [11].

Recent studies have shown that exosomal circRNAs have potential applications as disease biomarkers and novel therapeutic targets [12]. Comparatively, there have been few studies on the correlations between exosomal circRNAs and STEMI. Our study is based on the hypothesis that exosomal circRNAs can be used as potential biomarkers for STEMI. In the present study, we aim to identify differentially expressed exosomal circRNAs (DEECs) from microarray dataset (GSE160717 and GSE197137) and RNA-Seq dataset (GSE208194). The correlations between the expression levels of DEECs and clinicopathological features were analyzed, and the diagnostic and prognostic values of DEECs were evaluated. This approach may provide novel potential diagnostic and prognostic biomarkers for patients with STEMI.

## 2 Methods

### 2.1 Patients and plasma samples

The sample size of this study was calculated according to diagnostic accuracy studies [13,14]. A total of 128 participants aged from 18 to 75 years [64 STEMI patients (33 males and 31 females; average age of 48.60 ± 11.92 years) and 64 age- and gender-matched healthy controls (39 males and 25 females; average age of 50.14 ± 9.25 years)] were retrospective collected from Peking University Shenzhen Hospital (Shenzhen, China) between May 2017 and April 2019. The STEMI patients was defined according to the European Society of Cardiology guidelines [15], and the inclusion criteria were as follows: (1) patients with acute chest pain, chest tightness, and shortness of breath exceeded 30 min; (2) abnormal electrocardiogram with significant ST-segment elevation in at least two contiguous leads; (3) elevation of cTnI and CK-MB exceeded the upper 99th percentile of the reference values and exhibited dynamic evolution; and (4) the diagnostic coronary angiography confirmed coronary artery stenosis of more than 30%. The exclusion criteria were as follows: (1) patients with a previous history of cardiac diseases (myocardial infarction, valve abnormalities, cardiomyopathy, arrhythmia, and heart failure); (2) patients received percutaneous coronary intervention (PCI), venous thrombolytic injection, and anticoagulant within the past 6 months; (3) significant hepatic, renal, and respiratory failure, coagulation disorders, thyroid dysfunction, acute and chronic infection, immune system abnormalities, and malignant tumors; (4) pregnant women and patients with mental illnesses; and (5) patients with failed follow-up. The healthy controls with no clinical signs of acute chest pain, chest tightness and shortness of breath, and normal coronary arteries.

The patients were admitted to our hospital within 4 h after onset of clinical symptoms, and venous blood samples (5 mL) were collected before any medical treatment was implemented. The plasma was separated from venous blood after centrifuged at 3,000 rpm for 10 min at 4°C and was then stored at −80°C until exosomes isolation was performed. The research was approved by the Ethics Committee of Peking University Shenzhen Hospital. Written informed content was obtained from all participants.

### 2.2 Clinical indicators

The baseline data of STEMI patients and healthy controls were registered after admission, including age, gender, body mass index (BMI), and medical history (diabetes mellitus and hypertension). The laboratory indicators, including white blood cells (WBC), hemoglobin, total cholesterol (TC), triglyceride (TG), low density lipoprotein cholesterol (LDL-C), high density lipoprotein cholesterol (HDL-C), creatinine (CR), uric acid (UA), high sensitivity C-reactive protein (hs-CRP), lipoprotein a [LP(a)], cTnI, CK-MB, and N-terminal probrain natriuretic peptide (NT-proBNP), were detected by routine methods according to the manufacturer’s protocols.

The cardiovascular outcomes of STEMI patients were evaluated using major adverse cardiovascular events (MACEs), including angina, heart failure, malignant arrhythmias, cardiac death, and rehospitalization, due to STEMI recurrence. The MACE-free survival rate in STEMI patients up to 1 year after discharge was obtained from follow-up outpatient review and phone communication.

### 2.3 Screening of DEECs in patients with myocardial infarction from GEO database

The microarray and RNA-Seq data were obtained from GEO database (http://www.ncbi.nlm.nih.gov/gds/). The microarray dataset (GSE160717 and GSE197137) and the RNA-Seq dataset (GSE208194) were selected for screening DEECs in patients with myocardial infarction. R version 3.6.2 software (https://www.r-project.org/) was used for further analysis, and “|Log2 (fold change)| >  1 and *P*-value  <  0.05” were defined as the criteria for screening.

### 2.4 Exosomes isolation

For each sample, 500 µL of plasma was independently filtered with a 0.22 µm filter (Corning Incorporated, Corning, USA) and then exosomes were isolated using a UR52136 Exosome Isolation and Purification Kit (Umibio, Shanghai, China), according to the manufacturer’s protocol. After that, one of the exosomes was used for characteristic analysis via a transmission electron microscope (FEI Tecnai G2 T20; Thermo Fischer Scientific, Waltham, USA) operated at 120 kV, and the other was used for RNA extraction.

### 2.5 Exosomal RNA extraction and RNase R treatment

Total RNA of the acquired exosomes in each sample was extracted using a RNeasy mini kit (QIAGEN, Hilden, Germany), according to the manufacturer’s protocol. The concentrations of total RNA more than 100 ng/µL and absorbance ratio more than 2.0 at A260/280 and A260/230 were defined as having sufficient quality and quantity for further experiments. After that, 5 μg of total RNA was incubated with 15 U of RNase R (Epicenter Biotechnologies, Shanghai, China) treatment at 37°C for 20 min to remove the linear RNA.

### 2.6 Reverse transcription and quantitative real-time PCR (qRT-PCR)

1 μg of digested RNA was reverse transcribed into complementary DNA with random primers using a GoScript™ Reverse Transcription System (Promega Corporation, Madison, USA), according to the manufacturer’s protocol. Then, qRT-PCR procedure was performed using a GoTaq^®^ 2-Step RT-qPCR System (Promega Corporation) on a Applied Biosystems 7500 Real-Time PCR instrument (Thermo Fisher Scientific). The reaction was designed as follows: 95°C for 10 min, followed by 40 cycles of 95°C for 10 s and 59°C for 45 s. The glyceraldehyde-3-phosphate dehydrogenase (GAPDH) gene was used as an internal control. The sequences of the primers are shown in Table A1. The relative expression level of each gene was calculated using the 2^−ΔΔCT^ method.

### 2.7 Statistical analysis

The data of statistical analysis were conducted using SPSS software 28.0 (SPSS, Chicago, USA) and GraphPad Prism software 6.0 (GraphPad Prism Software Inc, San Diego, USA). Each independent experiment was repeated 3 times, and data were expressed as the percentages or the mean ± standard deviation. Significance between two groups was analyzed using an independent sample *t*-test (two tailed). The correlations between the levels of the DEECs and clinical characteristics in STEMI patients were assessed by Spearman’s correlation test. The area under the curves (AUC), sensitivity, and specificity for the DEECs, cTnI, and CK-MB were determined using receiver operating characteristic (ROC) curves analysis to assess its diagnostic efficiency in differentiating STEMI patients from healthy controls. Kaplan–Meier method and log-rank test were used to compare the MACE-free 1-year survival rate in different groups based on the levels of the DEECs. The univariate and multivariate Cox regression model analyses were performed to confirm independent factors for prognosis. *P* values less than 0.05 were considered statistically significant.

## 3 Results

### 3.1 Comparison of clinical characteristics in STEMI patients and healthy controls

The clinical characteristics of STEMI patients and healthy controls are shown in Table 1. The prevalence of hypertension and the LDL-C, hs-CRP, cTnI, CK-MB, and NT-proBNP levels were higher in STEMI patients than that in healthy controls. The level of HDL-C in STEMI patients was lower compared with healthy controls. However, no significant differences were observed in age, gender, BMI, prevalence of diabetes mellitus, WBC, hemoglobin, TC, TG, CR, UA, and LP(a) levels in the two groups.

**Table 1:** The clinical characteristics of STEMI patients and healthy controls

Characteristics	Healthy controls (*n* = 64)	STEMI patients (*n* = 64)	*P* value
Age (years)	50.14 ± 9.25	48.60 ± 11.92	0.122
Gender (man/female)	39/25	33/31	0.285
BMI (kg/m^2^)	24.18 ± 1.30	23.59 ± 1.47	0.592
Hypertension (*n*, %)	12 (18.75)	39 (60.94)	<0.001
Diabetes mellitus (*n*, %)	15 (23.44)	20 (31.25)	0.321
WBC (×10^9^/L)	7.51 ± 3.09	7.23 ± 4.40	0.308
Hemoglobin (g/L)	116.44 ± 17.85	123.15 ± 18.38	0.173
TC (mmol/L)	4.37 ± 0.92	4.96 ± 1.13	0.061
TG (mmol/L)	1.18 ± 0.45	1.32 ± 0.66	0.115
LDL-C (mmol/L)	2.49 ± 0.73	3.88 ± 0.91	0.007
HDL-C (mmol/L)	1.27 ± 0.30	0.95 ± 0.28	0.029
CR (μmol/L)	70.46 ± 42.56	68.31 ± 40.50	0.518
UA (μmol/L)	278.22 ± 126.18	247.17 ± 106.35	0.052
hs-CRP (mg/L)	6.74 ± 2.08	52.81 ± 43.95	<0.001
LP(a) (mg/L)	183.46 ± 112.70	192.11 ± 120.62	0.106
cTnI (ng/L)	20.97 ± 12.14	454.96 ± 421.87	<0.001
CK-MB (ng/mL)	3.06 ± 1.79	44.23 ± 40.85	<0.001
NT-proBNP (pg/mL)	350.03 ± 149.26	1731.59 ± 1334.27	<0.001

STEMI: ST-segment elevation myocardial infarction; BMI: body mass index; WBC: white blood cells; TC: total cholesterol; TG: triglyceride; LDL-C: low density lipoprotein cholesterol; HDL-C: high density lipoprotein cholesterol; CR: creatinine; UA: uric acid; hs-CRP: high sensitivity C-reactive protein; LP(a): lipoprotein a; cTnI: cardiac troponin I; CK-MB: creatine kinase isoenzymes; NT-proBNP: N-terminal probrain natriuretic peptide.

### 3.2 Identification and characterization of 4 DEECs in patients with myocardial infarction from GEO database

**Figure 1:**
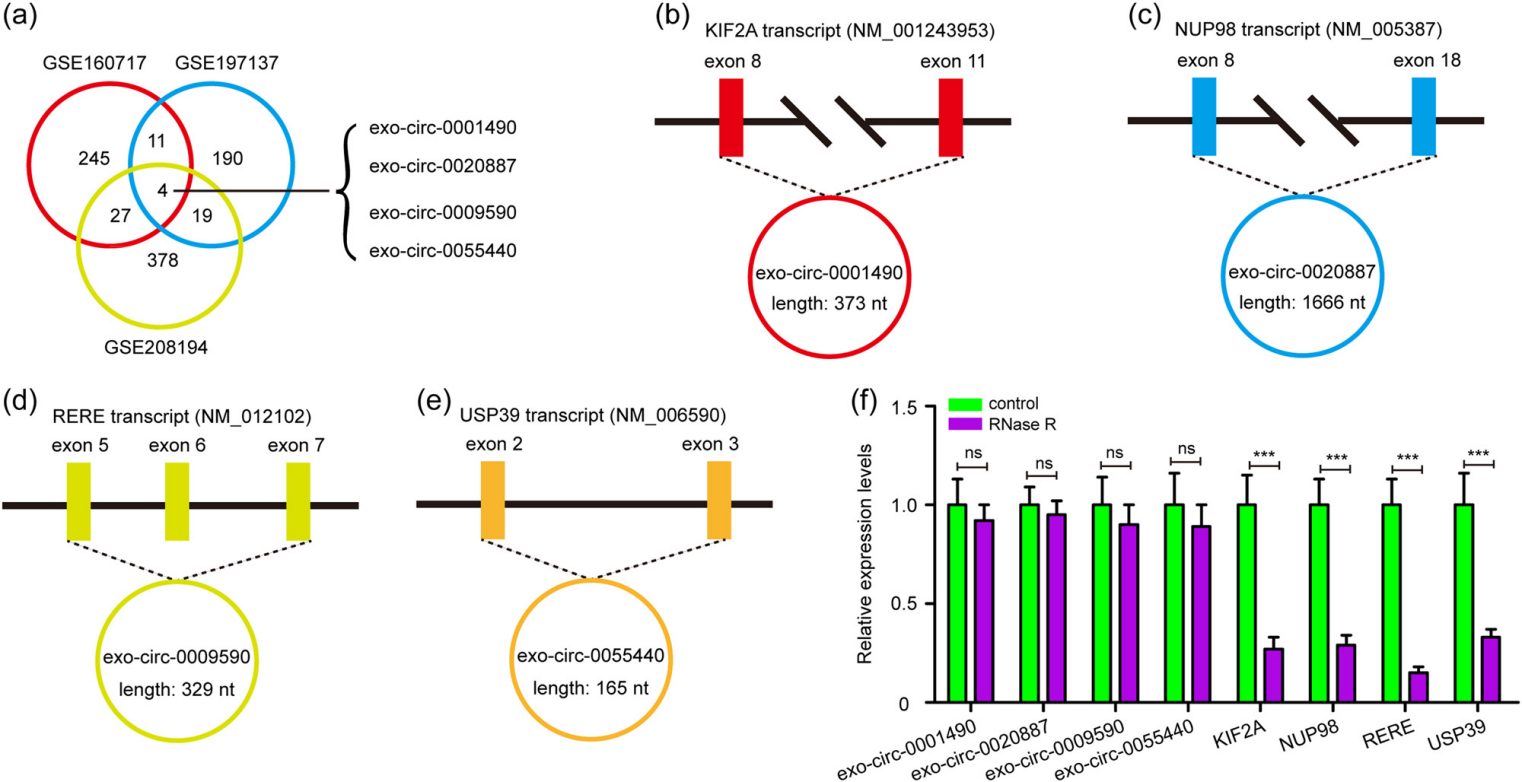
Identification and characterization 4 DEECs in patients with myocardial infarction from GEO database. (a) The microarray dataset (GSE160717 and GSE197137) and the RNA-Seq dataset (GSE208194) were downloaded for integrated analyses of DEECs, and venn diagram showed the intersection of DEECs. (b–e) The genomic loci and splicing schematic diagram of exo-circ-0001490, exo-circ-0020887, exo-circ-0009590, and exo-circ-0055440. (f) The stability of exo-circ-0001490, exo-circ-0020887, exo-circ-0009590, exo-circ-0055440, KIF2A, NUP98, RERE, and USP39 mRNA was detected by RNase R degradation assay. DEECs: differentially expressed exosomal circRNAs; KIF2A: kinesin heavy chain member 2A; NUP98: nucleoporin 98 and 96 precursor; RERE: arginine-glutamic acid dipeptide repeats; USP39: ubiquitin-specific peptidase 39; RNase R: Ribonuclease R. Data were expressed as mean ± standard deviation from three independent experiments. ^ns^ *P* > 0.05, ***P < 0.001.

A total of 287, 224, and 428 DEECs were identified in the microarray dataset (GSE160717 and GSE197137) and the RNA-Seq dataset (GSE208194), respectively. Furthermore, four DEECs were screened from the three cohorts, including exo-circ-0001490, exo-circ-0020887, exo-circ-0009590, and exo-circ-0055440 (Figure 1a).

The four DEECs were all exonic circRNAs, according to the annotation of circBank (http://www.circbank.cn/index.html) and circBase (http://www.circbase.org/). The genomic loci and splicing schematic diagram of exo-circ-0001490, exo-circ-0020887, exo-circ-0009590, and exo-circ-0055440 are shown in Figure 1b–e.

To further verify the circular characteristics of exo-circ-0001490, exo-circ-0020887, exo-circ-0009590, and exo-circ-0055440, we added RNase R to total RNA from plasma of STEMI patients, which can degrade linear RNAs characterized by a free 3′-terminus but have no influence on circRNAs. Results found that RNase R reduced the mRNA expression levels of kinesin heavy chain member 2A (KIF2A), nucleoporin 98 and 96 precursor (NUP98), arginine-glutamic acid dipeptide repeats (RERE), and ubiquitin-specific peptidase 39 (USP39), but have no significant effect on the expression levels of exo-circ-0001490, exo-circ-0020887, exo-circ-0009590, and exo-circ-0055440 (Figure 1f). The resistance to digestion via RNase R indicated that exo-circ-0001490, exo-circ-0020887, exo-circ-0009590, and exo-circ-0055440 have circular RNA structures.

### 3.3 Upregulated expression of exo-circ-0020887 and exo-circ-0009590 in plasma from STEMI patients

**Figure 2:**
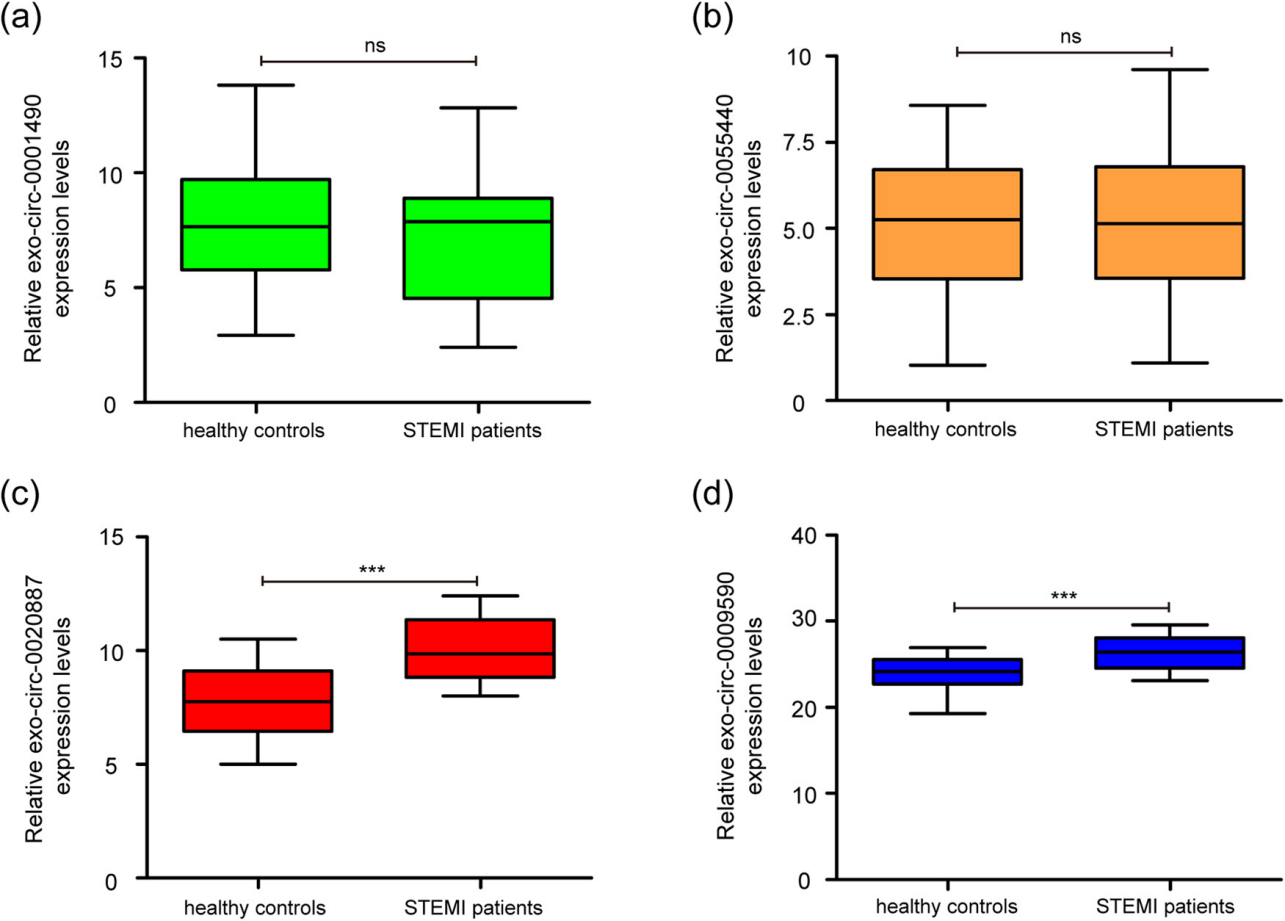
Upregulated expression of exo-circ-0020887 and exo-circ-0009590 in plasma from STEMI patients. (a and b) qRT-PCR analysis of exo-circ-0001490 and exo-circ-0055440 expression levels (normalized to GAPDH) in plasma from 64 STEMI patients and 64 healthy controls. (c and d) The expression levels of plasma exo-circ-0020887 and exo-circ-0009590 were increased in STEMI patients that in plasma of healthy controls. STEMI: ST-segment elevation myocardial infarction; qRT-PCR: quantitative real-time PCR. Data were expressed as mean ± standard deviation from three independent experiments. ^ns^ *P* > 0.05, ***P < 0.001.

The expression levels of exo-circ-0001490, exo-circ-0020887, exo-circ-0009590, and exo-circ-0055440 in STEMI patients were assessed using qRT-PCR. Among these circRNAs, there was no significant difference of exo-circ-0001490 and exo-circ-0055440 levels between the plasma from healthy controls and the plasma from STEMI patients (Figure 2a and b). Furthermore, exo-circ-0020887 expression in plasma of STEMI patients was upregulated than that in plasma of healthy controls (Figure 2c). Similarly, Figure 2d reveals that plasma from STEMI patients had higher exo-circ-0009590 levels when compared with that in plasma from healthy controls. Therefore, exo-circ-0020887 and exo-circ-0009590 were selected for subsequent investigation.

### 3.4 Correlations between plasma exo-circ-0020887 and exo-circ-0009590 levels and the clinical characteristics of STEMI patients

As shown in Table 2 and Table A2, the increased exo-circ-0020887 and exo-circ-0009590 expression was positively correlated with the levels of hs-CRP, LDL-C, cTnI, and CK-MB but was negatively correlated with LP(a) level. However, no associations were found between exo-circ-0020887 and exo-circ-0009590 levels with other clinicopathological features, including age, gender, BMI, diabetes mellitus and hypertension, WBC, hemoglobin, TC, TG, CR, UA, HDL-C, and NT-proBNP levels. These results suggested that upregulation of plasma exo-circ-0020887 and exo-circ-0009590 is associated with adverse clinical characteristics in STEMI patients.

**Table 2:** The correlations between the levels of exo-circ-0020887 and exo-circ-0009590 and clinical characteristics in 64 STEMI patients

Characteristics	Cases (*n* = 64)	Exo-circ-0020887 expression	*P* value	Exo-circ-0009590 expression	*P* value
Age (years)			0.721		0.598
<50	28	10.13 ± 1.67		26.72 ± 2.34	
≥50	36	9.93 ± 1.15		26.13 ± 2.16	
Gender			0.474		0.091
Man	33	9.86 ± 1.25		25.90 ± 2.08	
Female	31	10.19 ± 1.48		26.91 ± 1.96	
BMI (kg/m^2^)			0.954		0.617
<24	50	10.01 ± 1.70		26.50 ± 1.95	
≥24	14	10.05 ± 1.32		25.99 ± 1.82	
Hypertension			0.488		0.214
No	25	10.20 ± 1.34		26.86 ± 2.17	
Yes	39	9.90 ± 1.51		26.08 ± 2.05	
Diabetes mellitus			0.172		0.074
No	44	9.79 ± 1.55		26.05 ± 1.98	
Yes	20	10.52 ± 1.27		27.13 ± 2.34	
WBC (×10^9^/L)			0.309		0.673
<10	56	10.07 ± 1.54		26.32 ± 1.94	
≥10	8	9.67 ± 1.49		26.88 ± 2.53	
Hemoglobin (g/L)			0.327		0.542
<120	23	10.21 ± 1.23		25.97 ± 2.11	
≥120	41	9.91 ± 1.50		26.62 ± 1.99	
TC (mmol/L)			0.532		0.956
<5.69	47	9.96 ± 1.44		26.35 ± 1.92	
≥5.69	17	10.18 ± 1.59		26.50 ± 2.14	
TG (mmol/L)			0.475		0.125
<1.7	54	9.95 ± 1.30		26.25 ± 2.33	
≥1.7	10	10.39 ± 1.43		27.13 ± 2.10	
LDL-C (mmol/L)			0.006		<0.001
<3.4	22	8.81 ± 1.05		23.98 ± 1.63	
≥3.4	42	10.65 ± 1.39		27.65 ± 2.29	
HDL-C (mmol/L)			0.136		0.085
<1.08	30	9.77 ± 1.32		26.94 ± 2.19	
≥1.08	34	10.24 ± 1.47		25.90 ± 2.26	
CR (μmol/L)			0.851		0.712
<106	61	10.02 ± 1.55		26.40 ± 2.45	
≥106	3	9.96 ± 1.20		26.18 ± 2.39	
UA (μmol/L)			0.429		0.529
<300	49	9.90 ± 1.25		26.22 ± 2.17	
≥300	15	10.41 ± 1.38		26.94 ± 1.98	
hs-CRP (mg/L)			0.015		<0.001
<10	12	9.15 ± 0.95		23.52 ± 1.58	
≥10	52	10.22 ± 1.63		27.05 ± 2.07	
LP(a) (mg/L)			<0.001		<0.001
<180	35	11.13 ± 1.05		27.24 ± 2.15	
≥180	29	8.68 ± 1.21		25.36 ± 2.27	
cTnI (ng/L)			0.011		0.035
<40	16	9.03 ± 1.40		25.31 ± 2.23	
≥40	48	10.35 ± 1.19		26.75 ± 2.45	
CK-MB (ng/mL)			0.027		0.030
<5	20	9.24 ± 1.17		25.42 ± 2.20	
≥5	44	10.37 ± 1.65		26.83 ± 2.09	
NT-proBNP (pg/mL)			0.473		0.971
<450	6	10.31 ± 1.38		26.33 ± 2.28	
≥450	58	9.99 ± 1.26		26.39 ± 2.43	

Exo-circ-0020887: exosomal circular RNA 0020887; exo-circ-0009590: exosomal circular RNA 0009590.

### 3.5 Diagnostic efficacy of plasma exo-circ-0020887 and exo-circ-0009590 for STEMI patients

**Figure 3:**
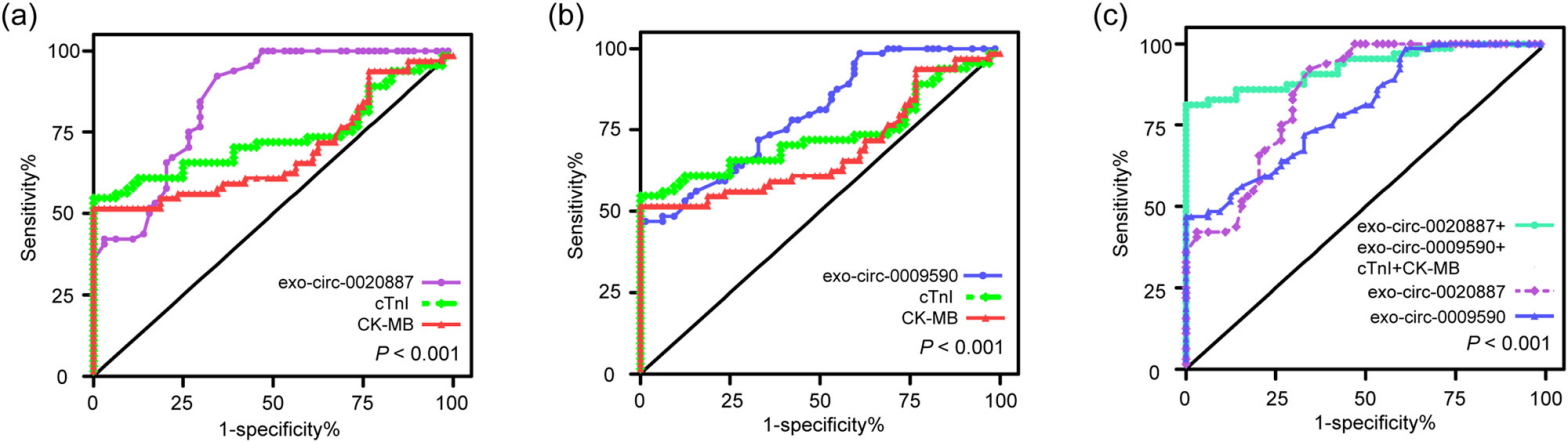
Diagnostic efficacy of plasma exo-circ-0020887 and exo-circ-0009590 in STEMI patients. (a) ROC curves analysis of exo-circ-0020887, cTnI, and CK-MB for differentiating STEMI patients from healthy controls. (b) ROC curves analysis showed that the diagnostic power of exo-circ-0009590 for STEMI exceeded cTnI and CK-MB. (c) The exo-circ-0020887 and exo-circ-0009590 in combination with cTnI and CK-MB had the highest diagnostic accuracy. ROC: receiver operating characteristic; cTnI: cardiac troponin I; CK-MB: creatine kinase isoenzymes.

To further verify the efficacy of exo-circ-0020887 and exo-circ-0009590 for the diagnosis of STEMI, ROC curves were plotted and compared with cTnI and CK-MB. Figure 3a reveals that plasma exo-circ-0020887 had greater AUC (0.85 vs 0.73 vs 0.69), sensitivity (84.38% vs 70.31% vs 59.38%), and specificity (70.33% vs 60.94% vs 60.70%) values than cTnI and CK-MB in diagnosing STEMI within 4 h after onset. Similarly, the diagnostic efficiency of exo-circ-0009590 for STEMI early detection was higher than cTnI and CK-MB, according to the AUC (0.80 vs 0.73 vs 0.69), sensitivity (71.88% vs 70.31% vs 59.38%), and specificity (67.19% vs 60.94% vs 60.70%) values (Figure 3b). Moreover, when exo-circ-0020887 and exo-circ-0009590 combined with cTnI and CK-MB, the diagnostic value was greatly improved compared with anyone of them (AUC: 0.93, sensitivity: 81.25%, specificity: 98.44%) (Figure 3c). Therefore, we concluded that plasma exo-circ-0020887 and exo-circ-0009590 might be used as potential biomarkers for STEMI early detection and could improve the diagnostic efficacy when in combined with cTnI and CK-MB.

### 3.6 Prognostic values of plasma exo-circ-0020887 and exo-circ-0009590 in STEMI patients

**Figure 4:**
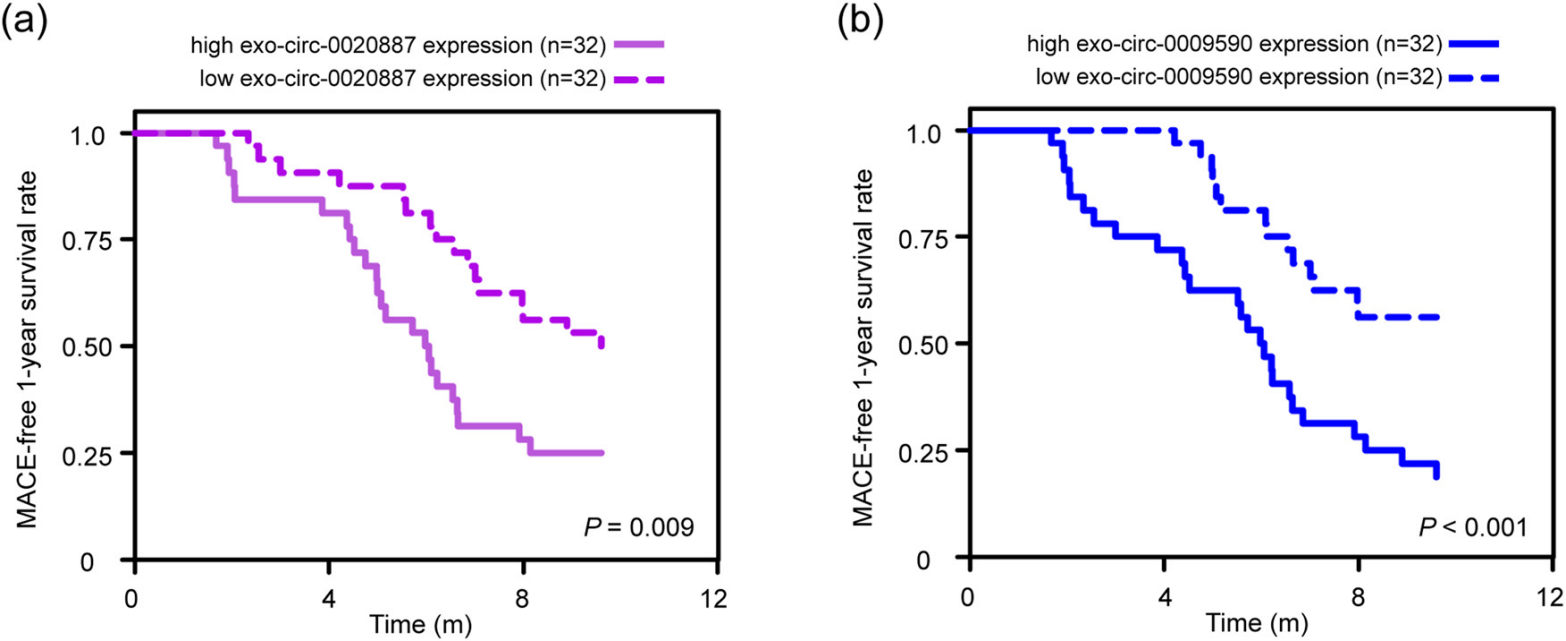
Prognostic value of plasma exo-circ-0020887 and exo-circ-0009590 in STEMI patients. (a) Kaplan–Meier survival curves analysis of the MACE-free 1-year survival rate in STEMI patients based on exo-circ-0020887 expression status. (b) The MACE-free 1-year survival rate was lower in STEMI patients with high exo-circ-0009590 expression than those patients with low exo-circ-0009590 expression. MACE: major adverse cardiovascular events.

According to the median value (9.84) of exo-circ-0020887 expression, 64 STEMI patients were divided by high exo-circ-0020887 expression group (*n* = 32) and low exo-circ-0020887 expression group (*n* = 32). Similarly, based on the median value (26.45) of exo-circ-0009590 expression, 64 STEMI patients were classified into the high exo-circ-0009590 expression group (*n* = 32) and the low exo-circ-0009590 expression group (*n* = 32). Kaplan–Meier analysis showed that MACE-free 1-year survival rate in STEMI patients with high exo-circ-0020887 expression was lower than that in those patients with low exo-circ-0020887 expression (Figure 4a). Similar results of lower MACE-free survival rate were found in STEMI patients with high exo-circ-0009590 expression when compared with low exo-circ-0009590 expression (Figure 4b).

**Figure A1:**
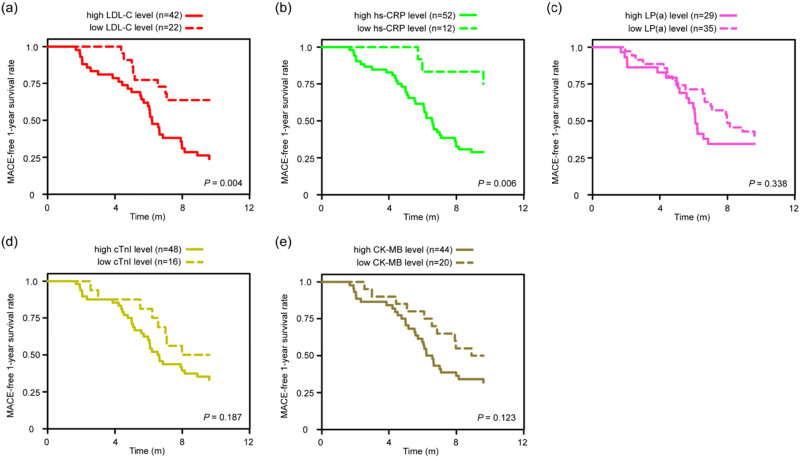
Correlations between the MACE-free 1-year survival rate and LDL-C, hs-CRP, LP(a), cTnI and CK-MB expression in STEMI patients. (a–e) Kaplan-Meier survival curves analysis of the MACE-free 1-year survival rate in STEMI patients based on the levels of LDL-C, hs-CRP, LP(a), cTnI and CK-MB. MACE: major adverse cardiovascular events; LDL-C: low density lipoprotein cholesterol; hs-CRP: high sensitivity C-reactive protein; LP(a): lipoprotein a; cTnI: cardiac troponin I; CK-MB: creatine kinase isoenzymes.

Additionally, we analyzed the correlations of the levels of LDL-C, hs-CRP, LP(a), cTnI, and CK-MB with prognosis in STEMI patients. The results revealed that high hs-CRP expression and high LDL-C expression were associated with lower MACE-free 1-year survival rate (Figure A1a and b). And no correlation of LP(a), cTnI, and CK-MB levels with MACE-free 1-year survival rate was found (Figure A1c–e). These results demonstrated that elevated plasma exo-circ-0020887, exo-circ-0009590, hs-CRP, and LDL-C predicted short-term adverse cardiovascular outcomes.

### 3.7 Factors affecting the short-term cardiovascular outcomes of STEMI patients

As shown in Table A3, the results from univariate Cox regression analysis regarding the clinical variables and their impact on the MACE-free 1-year survival rate of STEMI patients, indicating that hypertension, WBC, LDL-C, hs-CRP, exo-circ-0020887 and exo-circ-0009590 expression were risk factors for short-term adverse cardiovascular outcomes. In addition, multivariate Cox regression analysis showed that hypertension, hs-CRP, exo-circ-0020887, and exo-circ-0009590 expression were independent risk prognosticators. Together, elevated plasma exo-circ-0020887 and exo-circ-0009590 might used as biomarkers for short-term adverse cardiovascular outcomes of STEMI patients.

## 4 Discussion

The cTnI and CK-MB are the most common biomarkers for STEMI diagnosis, but their utilizing may be limited in some cases due to hysteresis, non-specificity, and other factors. Therefore, researchers are currently investigating novel promising biomarkers for STEMI diagnosis and prognosis with use in clinical practice. In consideration of their roles, steadily growing studies in life science of the expression pattern reveal that ncRNAs are dysregulated in venous blood, showing their potential as biomarkers for STEMI [16,17]. For example, Salama et al. reported that miR-208a is a good diagnostic biomarker and a predictor of no-reflow in STEMI patients undergoing primary PCI [18]. Xiao et al. indicated that miR-146a acts as a biomarker for adverse prognosis of STEMI patients [19]. In a recent study by Horváth et al. showed that miR-331 and miR-151-3p are novel biomarkers in STEMI and may be associated with plaque rupture [20]. In another study by Yang et al. suggested that lncRNA MALAT1 could serve as an effective biomarker of no-reflow phenomenon in STEMI patients receiving primary PCI [21]. In addition, highly expressed lncRNA LIPCAR in plasma serves as a warning sign for the diagnosis of STEMI [22]. In this study, we identified exo-circ-0020887 and exo-circ-0009590 in patients with myocardial infarction from GEO database. The exo-circ-0020887 and exo-circ-0009590 expression was upregulated in STEMI patients and was positively correlated with hs-CRP, LDL-C, cTnI, and CK-MB. Elevated plasma exo-circ-0020887 and exo-circ-0009590 had a good diagnostic efficacy for STEMI and predicted short-term adverse cardiovascular outcomes.

Previous studies have confirmed that circRNAs can be stably and repeatedly detected in exosomes [23]. Thus, the specific expression of exo-circRNAs could be a promising biomarker for many diseases. Regarding myocardial infarction, a study of high-throughput RNA sequencing found that 3,862 circRNAs are dysregulated in peripheral blood, and the differentially expressed circ-TMEM165, circ-UBAC2, circ-ZNF609, circ-ANKRD12, and circ-SLC8A1 are confirmed in the AC16 cell model [24]. Also, Yin et al. identified 650 circRNAs, including 115 downregulated circRNAs and 535 upregulated circRNAs, that are differentially expressed in plasma from AMI patients [25].

The exo-circ-0020887 and exo-circ-0009590 are two novel circRNAs, which were chose following the microarray dataset (GSE160717 and GSE197137) and the RNA-Seq dataset (GSE208194). To date, the researches about specific connections between exo-circ-0020887 and exo-circ-0009590 levels and presence of STEMI and its prognostic value are unclear. Here, we found that the expression levels of exo-circ-0020887 and exo-circ-0009590 were increased in plasma from STEMI patients. Meanwhile, a positive correlation was observed between upregulated exo-circ-0020887 and exo-circ-0009590 expression and hs-CRP, LDL-C, cTnI, and CK-MB levels. More importantly, we found that the diagnostic power of exo-circ-0020887 and exo-circ-0009590 for STEMI exceeded the common markers cTnI and CK-MB and could therefore be used as biomarkers to improve the clinical diagnosis of STEMI.

Moreover, based on the Kaplan–Meier method, we showed that STEMI patients with high levels of exo-circ-0020887 and exo-circ-0009590 had lower MACE-free survival rate than those patients with low exo-circ-0020887 and exo-circ-0009590 levels during the 1-year follow-up period. Additionally, we found that high hs-CRP expression and high LDL-C expression were associated with lower MACE-free 1-year survival rate. Hypertension is traditional risk factor for MACE-free survival rate [26]. The MACE-free survival rate of STEMI is affected by inflammation and WBC, and LDL-C plays an important role in mediating the release of inflammatory cytokines [27]. Consistent with previous studies [19,28], we found that hypertension, WBC, LDL-C, hs-CRP, exo-circ-0020887, and exo-circ-0009590 expression were risk factors for short-term adverse cardiovascular outcomes in univariate Cox regression model. Interestingly, multivariate Cox regression model showed that hypertension, hs-CRP, exo-circ-0020887, and exo-circ-0009590 expression were independent risk prognosticators. Thus, plasma increased exo-circ-0020887 and exo-circ-0009590 might prove to be useful biomarkers for the prediction of short-term adverse cardiovascular outcomes.

Growing evidence have confirmed that circRNAs performing its function by acting as a “sponging” to miRNAs [29]. We used circRNAs interactome tool (https://circinteractome.nia.nih.gov/index.html) to find that several miRNAs could be targeting by exo-circ-0020887 and exo-circ-0009590, including miR-330-3p, miR-637, miR-661, miR-1236, and miR-1299. Among these miRNAs, previous studies revealed that the biological processes of miR-637 and miR-1299 are related to vascular endothelial cell dysfunction and vascular smooth muscle cell injury, respectively [30,31]. Accordingly, we speculated that exo-circ-0020887 and exo-circ-0009590 might be involved in the development of STEMI by sponge of miR-637 and miR-1299.

The limitation of our research is that a relatively small sample size was used, and further investigation with a larger sample size with a longer follow-up study is required to obtain accurate and reproducible results. In addition, the function and molecular mechanism of exo-circ-0020887 and exo-circ-0009590 in STEMI still need further exploration.

## 5 Conclusion

In conclusion, our research demonstrated that the expression levels of plasma exo-circ-0020887 and exo-circ-0009590 are upregulated in STEMI patients and might serve as promising biomarkers for the diagnosis and prediction of short-term adverse cardiovascular outcomes of STEMI.

## Appendix

**Table A1:** Sequences of primers for qRT-PCR

Name	Sequences	
exo-circ-0001490	Forward	5′-CAGGAAAGAACCAAGATTGTTCTAA-3′
	Reverse	5′-GTACCATCACAACATCTTTACTAGG-3′
exo-circ-0020887	Forward	5′-TGGATGATATTGTGCATATCCGGA-3′
	Reverse	5′-TCTGGGTGGTTGTAGCCTG-3′
exo-circ-0009590	Forward	5′-TGTCATTACAGACCCAGTTATCA-3′
	Reverse	5′-AGATGCTGTGGTGGCTGT-3′
exo-circ-0055440	Forward	5′-CTGTGTTCTATCTCCCTCTCAC-3′
	Reverse	5′-TGTCCAGGTACGGGCAGT-3′
KIF2A	Forward	5′-AAGGCAAAGAGATTGACCTGG-3′
	Reverse	5′-GAAGCTACAGTCCGTCGATTC-3′
NUP98	Forward	5′-GGAATCCGATGTCAGACCCTAA-3′
	Reverse	5′-TTGGCTGTGCCTGTTGTTTGTA-3′
RERE	Forward	5′-AGGGTTTGCAGGGACGATGTTA-3′
	Reverse	5′-AATGGAAGAGGTCTGATGGAGC-3′
USP39	Forward	5′-CACTTACCTGCCGGGTATTGT-3′
	Reverse	5′-CCTGGAGGACGTTTGATGTTCT-3′
GAPDH	Forward	5′-ATCTTCCAGGAGCGAGATCCC-3′
	Reverse	5′-TGAGTCCTTCCACGATACCAA-3′

KIF2A: kinesin heavy chain member 2A; NUP98: nucleoporin 98 and 96 precursor; RERE: arginine-glutamic acid dipeptide repeats; USP39: ubiquitin specific peptidase 39; GAPDH: glyceraldehyde-3-phosphate dehydrogenase.

**Table A2:** The correlation coefficient between exo-circ-0020887 and exo-circ-0009590 expression and the levels of hs-CRP, LDL-C, LP(a), cTnI, and CK-MB

Indicators	exo-circ-0020887		exo-circ-0009590	
	*r*	*P* value	*r*	*P* value
hs-CRP	0.386	0.025	0.622	<0.001
LDL-C	0.770	<0.001	0.403	0.027
LP(a)	-0.491	0.004	-0.312	0.046
cTnI	0.516	<0.001	0.445	0.020
CK-MB	0.845	<0.001	0.757	<0.001

**Table A3:** Univariate and multivariate Cox regression model analysis of the factors affecting the MACE-free 1-year survival rate of STEMI patients

Characteristics	Univariate HR (95% CI)	*P* value	Multivariate HR (95% CI)	*P* value
Age (≥50 vs <50)	0.782 (0.214–2.393)	0.584	NA	NA
Gender (female vs man)	1.147 (0.576–2.737)	0.162	NA	NA
BMI (≥24 vs <24)	0.753 (0.145–1.329)	0.722	NA	NA
Hypertension (Yes vs No)	3.018 (1.986–9.255)	<0.001	2.659 (1.457–5.864)	0.002
Diabetes mellitus (Yes vs No)	1.114 (0.291–3.051)	0.214	NA	NA
WBC (≥10 vs <10)	2.149 (1.264–4.831)	0.015	1.395 (0.528–2.906)	0.124
Hemoglobin (≥120 vs <120)	0.611 (0.187–1.598)	0.620	NA	NA
TC (≥5.69 vs <5.69)	0.572 (0.251–1.345)	0.706	NA	NA
TG (≥1.7 vs <1.7)	1.806 (0.532–4.356)	0.193	NA	NA
LDL-C (≥3.4 vs <3.4)	2.520 (1.341–4.737)	0.007	2.013 (0.815–4.311)	0.059
HDL-C (≥1.08 vs <1.08)	1.701 (0.435–3.741)	0.087	NA	NA
CR (≥106 vs <106)	0.965 (0.451–1.663)	0.920	NA	NA
UA (≥300 vs <300)	1.593 (0.874–3.511)	0.088	NA	NA
hs-CRP (≥10 vs <10)	2.673 (1.341–5.437)	0.007	2.015 (1.178–4.751)	0.038
LP(a) (≥180 vs <180)	1.364 (0.722–2.576)	0.231	NA	NA
cTnI (≥40 vs <40)	1.581 (0.801–3.124)	0.150	NA	NA
CK-MB (≥5 vs <5)	1.660 (0.871–3.163)	0.102	NA	NA
NT-proBNP (≥450 vs <450)	1.037 (0.651–2.808)	0.469	NA	NA
Exo-circ-0020887 (high vs low)	2.339 (1.235–4.427)	0.005	2.422 (1.624–4.948)	<0.001
Exo-circ-0009590 (high vs low)	3.001 (1.583–5.685)	<0.001	2.792 (1.549–5.273)	0.002

HR: hazard ratio; CI: confidence interval; NA: not available.
